# Unravelling the phenotype of cardiovascular inflammation with magnetic resonance imaging: detecting the change with anti-TNF treatment in patients with rheumatoid arthritis

**DOI:** 10.1186/1532-429X-13-S1-P320

**Published:** 2011-02-02

**Authors:** Valentina O Puntmann, Peter Taylor, Rolf Gebker, Bernhard Schnackenburg, Amedeo Chiribiri, Andreas Schuster, Eike Nagel

**Affiliations:** 1 King's College London, Division of Imaging Sciences and Biomedical Engineering/The German Heart Institute Berlin, London, UK; 2 Imperial College London, The Kennedy Institute, London, UK; 3 The German Heart Institute Berlin, Berlin, Germany; 4 King's College London, Division of Imaging Sciences and Biomedical Engineering, London, UK

## Objective

To investigate the phenotypic relationships between aortic stiffness and left ventricular (LV) modes of deformation in patients with rheumatoid arthritis (RA) with anti-TNF therapy.

## Background

Excess of cardiovascular (CV) morbidity and mortality in RA patients is not fully explained by traditional CV risk factors. Evidence suggests the presence of inflammation-induced vascular and myocardial injury and dysfunction, leading to premature atherosclerosis and heart failure. Aortic stiffness, an independent predictor of cardiac events and a marker of pulsatile LV afterload, improves with anti-tumor necrosis factor-alpha (anti-TNF) therapy in these patients; however, the effects on determinants of systolic function remain undetermined.

## Methods

**Figure 1:**
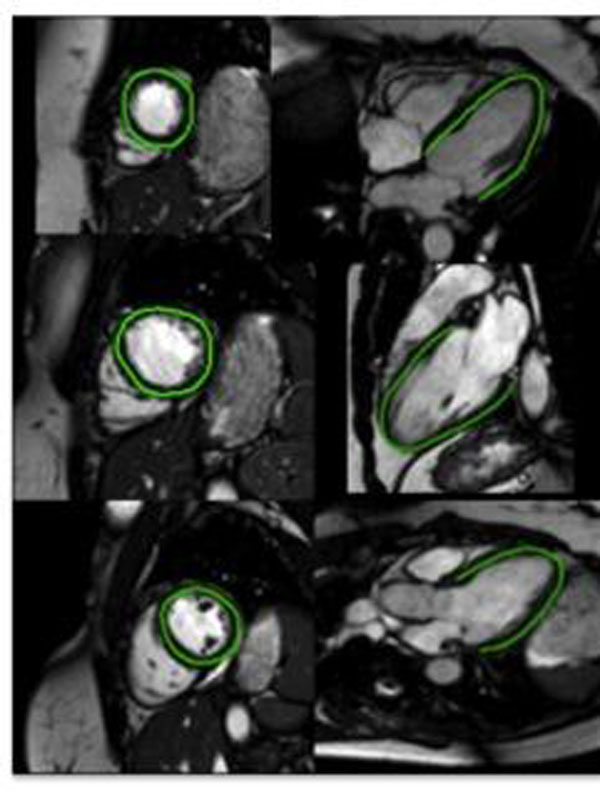


Of the total of 24 patients with RA, free of cardiac symptoms, thirteen subjects, eligible for anti-TNF therapy as per its licensed indications, completed paired standardized MRI assessments of aortic pulse wave velocity (PWV), global LV function and deformation (peak systolic strains and rotation Figure 1), before initiation and after 3-month of therapy. To avoid confounding with a possible ischaemic scar, late gadolinium enhancement was also performed.

## Results

**Figure 2:**
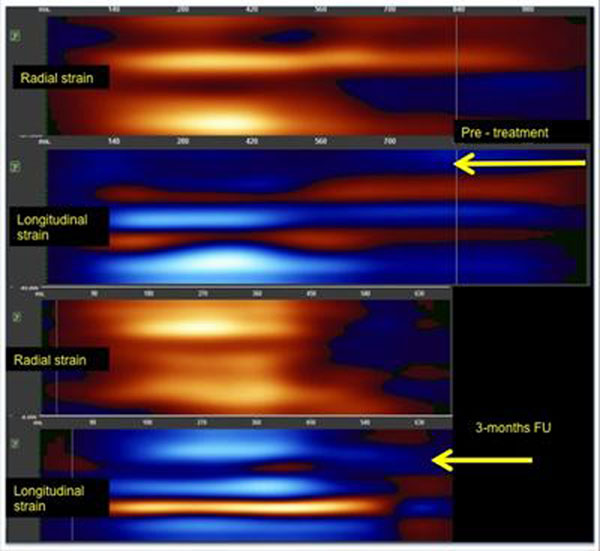


At the follow up, the treated group with mean age 42.221.1 years and female predominance (n=8), showed reduced disease activity score and CRP (mean difference SD): 32.447.8 mg/L, p<0.01), reduced aortic PWV (0.75±0.32 m/s, p<0.01). Despite reduced LV end-diastolic volume (EDV: 9.78.7ml, p<0.01) and increased stroke volume (SV: 5.13.8ml, p=0.02), ejection fraction was no different from the baseline (EF: 0.085.1%, p=0.3). LV rotation rate significantly increased, more pronounced in the apex than the base (rotation rate AP: -4.63.7/sec vs. rotation rate BA: -2.12.5/sec), corresponding with a greater improvement in apical radial strain (AP: -3.22.3 vs. BA: -2.23.2) and total longitudinal strain (long: 4.54.9) (Figure 2). In multiple regression analyses (stepwise), baseline aortic PWV correlated independently with log-transformed CRP and radial apical strain (R2=0.973, p<0.001), whereas, after 3 months of therapy, aortic PWV correlated with radial and circumferential apical and total longitudinal strain (R2 = 0.93, p<0.001). Measures of global systolic function showed no associations with aortic stiffness.

## Conclusion

Targeted anti-inflammatory therapy, such as with anti-TNF, is associated with improvement in aortic stiffness and myocardial deformation in RA patients with high-grade systemic inflammation. Our findings suggest that CV dysfunction in these patients is inflammation-induced and reversible. Our results may indicate a discernible CV phenotype by means of CMR, which can be accounted for by CV inflammation, and potentially instrumental as a guide for new anti-inflammatory interventions.

